# Study of Three Interface Pressure Measurement Systems Used in the Treatment of Venous Disease

**DOI:** 10.3390/s20205777

**Published:** 2020-10-12

**Authors:** Gayani K. Nandasiri, Arash M. Shahidi, Tilak Dias

**Affiliations:** Advanced Textiles Research Group, School of Art and Design, Nottingham Trent University, Nottingham NG1 4GG, UK; arash.shahidi@ntu.ac.uk (A.M.S.); tilak.dias@ntu.ac.uk (T.D.)

**Keywords:** pressure sensor, compression therapy, interface pressure measurement, sensor evaluation, flexible piezoresistive pressure sensors

## Abstract

The aim of the publication is to report the accuracy, repeatability and the linearity of three commercially available interface pressure measurement systems employed in the treatment of venous disease. The advances in the treatment and management of chronic venous disease by compression therapy have led to considerable research interest in interface pressure measurement systems capable of measuring low-pressure ranges (10–60 mmHg). The application of a graduated pressure profile is key for the treatment of chronic venous disease which is achieved by using compression bandages or stockings; the required pressure profiles are defined in standards (BSI, RAL-GZ, or AFNOR) for different conditions. However, achieving the recommended pressure levels and its accuracy is still deemed to be a challenge. Thus, it is vital to choose a suitable pressure measurement system with high accuracy of interface pressure. The authors investigated the sensing performance of three commercially available different pressure sensors: two pneumatic based (AMI and PicoPress^®^) and one piezoresistive (FlexiForce^®^) pressure sensors, with extensive experimental work on their performance in terms of linearity, repeatability, and accuracy. Both pneumatic based pressure measurement systems have shown higher accuracy in comparison to the flexible piezoresistive pressure sensors.

## 1. Introduction

Compression therapy is the most widely used treatment method for preventing adverse effects of chronic venous disease (CVD) [1,2]. CVD, including varicose veins and chronic venous insufficiency, affects the quality of life of many individuals around the world [1,3,4,5]. The compression therapy aims to compress the veins in order to increase the blood flow velocity and improve valvular function providing better coaptation of valve cusps. The current approach for compression therapy takes the form of conventional passive systems such as medical compression bandages, graduated compression stockings, and active systems such as intermittent pneumatic compression devices [1,3].

The accurate interface pressure measurement is considered as one of the most challenging tasks faced in the fields of medical and biomedical engineering. Its importance also extends to fields like sports science, management of diabetes-related ulcers, prosthetics, and human joint studies [2,3,4,5,6]. The measurement of the interface pressure applied by the compression devices (bandage systems, stockings) are required for reasons such as the efficiency of the compression therapy product for the treatment of venous disease, to evaluate the applied pressure by medical compression bandages to improve the training of the bandaging technique, to design better compression systems and also to study the effect of different postures of patients on the applied pressures. Hence, different standards (BSI, RAL-GZ, AFNOR) were formulated to outline the graduated pressure requirements (class Ⅰ, Ⅱ, Ⅲ, and Ⅳ), which signify the graduated pressure requirements for the areas ankle-to-midcalf or ankle-to-below-knee. The research on active compression since 2015 has developed compression therapy to be enhanced towards self-controllability and dynamic compression [6]. In such future developments of self-controllable active compression solutions, it would be necessary to measure and record the accurate pressure values to evaluate the performance.

There are many categories of direct interface pressure measuring systems available such as Kikuhime^®^ [7,8,9,10,11], SIGat-Tester^®^ [12], PicoPress^®^ [12,13], AMI [1,14], and indirect pressure measuring systems such as flexible piezoresistive force sensors [15,16,17] and Pliance X^®^ [18]. Pneumatic based systems such as Kikuhime^®^, PicoPress^®^, and AMI are the most common type of pressure measurement systems used in measuring compression pressure in clinical practice, which inherit the advantages of having flexible and thin probes. Partsch and Mosti [19] compared PicoPress^®^ to Kikuhime^®^ and SIGat-Tester^®^ and determined PicoPress^®^ was the most accurate with the least variation. However, they have not tested for pressures lower than 10 mmHg. Comparatively, there is less information available about both the static and dynamic performance of AMI in comparison with the other pressure measurement systems.

There have been previous studies carried out on these available flexible piezoresistive force sensors in determining the applied pressure during the compression therapy-based treatments for CVD [7,15,20,21]. These force sensors consist of a force sensing piezoresistive material with electrical resistance properties that vary with the applied force, thus the interface pressure is calculated measuring the applied force per unit area. The most widely used commercially available sensors under this category are the Interlink FSR (Interlink Electronics Inc., Camarillo, CA, USA) and Tekscan FlexiForce^®^ (Tekscan Inc., South Boston, MA, USA). Force sensing resistors (FSRs) are considered suitable for medical applications due to their thin, flexible nature, small size, and easy integration into the textiles [22].

However, only a few studies have reported on the accuracy and repeatability of these force sensors (indirect pressure measuring system) against the other available pressure measurement systems (direct) used for compression therapy. Also, there have been conflicting results on these piezoresistive sensors; Al Khaburi et al. [23] indicated that FlexiForce^®^ sensors have less accuracy compared to the PicoPress^®^, where Chi et al. [24] indicated that the piezoresistive sensors have shown similar in-vitro performance as PicoPress^®^. Therefore, it is difficult to draw conclusions on the suitability of the FSR pressure measurement systems for compression therapy applications.

This article presents a comparative performance evaluation of the most widely used three pressure measurement systems in compression therapy. The pressure measurement systems; AMI, PicoPress^®^, and FlexiForce^®^ were evaluated against a liquid manometer which is considered as the gold standard for the pressure measurement. The experiments were conducted in the pressure ranges of 0–90 mmHg which is considered as the appropriate interface pressure region in the compression therapy. The performance parameters required for effective validation of the interface pressure measurement systems such as repeatability, linearity, hysteresis, and accuracy were compared against each interface pressure measurement sensors.

## 2. Materials and Methods

The sensors of the pressure measurement systems (PMS) were calibrated in accordance with the manufacturer’s instructions; calibration can be identified as a process of comparing the output of a measurement system against a standard of known accuracy [25]. Different pressure sensors have different calibration procedures specified by the manufacturers hence, each sensor was calibrated separately in order to provide a reliable platform to study the accuracy, linearity, and repeatability of the sensors.

### 2.1. Sensors

**Figure 1:**
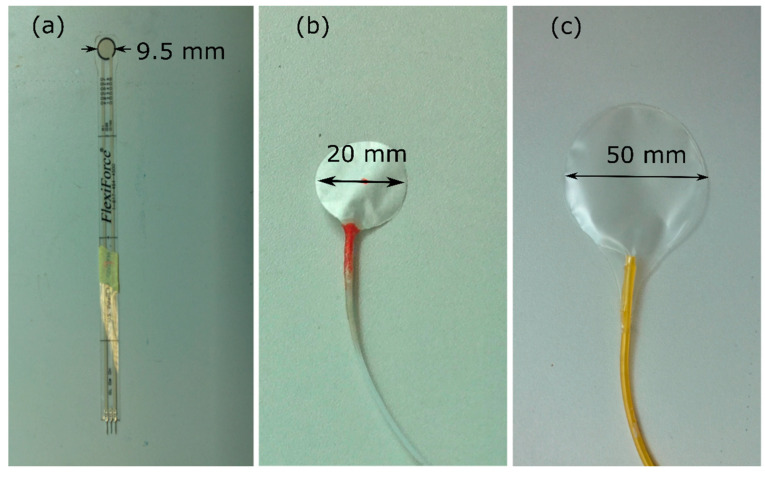
The sensors used for the evaluation (**a**) FlexiForce^®^ sensor (**b**) AMI air-pack sensor (**c**) PicoPress^®^ sensor.

Three commercially available interface pressure measurement systems, FlexiForce^®^ (Tekscan Inc., Boston, MA, USA), AMI air-pack sensors (AMI Techno, Tokyo, Japan), and PicoPress^®^ (Microlab Electronica, Padua, Italy) were used in the study. FlexiForce^®^ sensors (Figure 1a) are of 0.203 mm in thickness, 9.53 mm in diameter, and are constructed using two thin polyester films with one side of the films applied with a silver conductive material. A defined quantity of a pressure-sensitive ink material is sandwiched between them to form a pressure-sensitive area [17,25]. Two adhesive layers are used to laminate the two polyester films to form the sensor with the silver layers of the thin polyester films forming conductive leads. These sensors were connected to a CEBO-MSA 64 multi-sensor measurement box (CESYS GmbH, Herzogenaurach, Germany), and CEBO MSA lab (CESYS GmbH, Herzogenaurach, Germany) software was used to determine the pressure. The software can be used to calibrate and measure the pressure. In this research, eight FlexiForce^®^ sensors were procured from two different batches, with four sensors from each batch used for the evaluation.

AMI air-pack sensors (Figure 1b) are manufactured by AMI Techno, Tokyo, Japan having a thin sensor-bladder (1 mm thickness) of four diameters (15 mm, 20 mm, 25 mm, and 30 mm). However, a 20 mm diameter sensor, which is considered as a standard sensor for medical applications was used in this study. This pneumatic pressure measurement system consists of the main unit (model: AMI 0905-SA-35K) with a manually operated sensor-bladder inflation mechanism and a digital display; the unit has an auto-zero adjustment function. For this study, eight AMI air-pack sensor-bladders were procured that have been produced in two different batches, with four sensor-bladders from each batch. Both these sensor types were procured from two different batches in order to study any variations that may occur due to the batch-wise manufacturing.

PicoPress^®^ is a portable pneumatic pressure measuring system manufactured by Microlab Electronica, Padua, Italy. It has a sensor-bladder of 50 mm diameter and 3.0 mm thickness once inflated and 0.2 mm when deflated (Figure 1c). The device includes a manually operated pump to inflate a sensor-bladder and could be used to measure pressures in the range of 0−200 mmHg with a possibility of storing the recorded data in the device for dynamic pressure measurement; two PicoPress^®^ sensors were procured for this study.

### 2.2. Calibration of the Sensors

**Figure 2:**
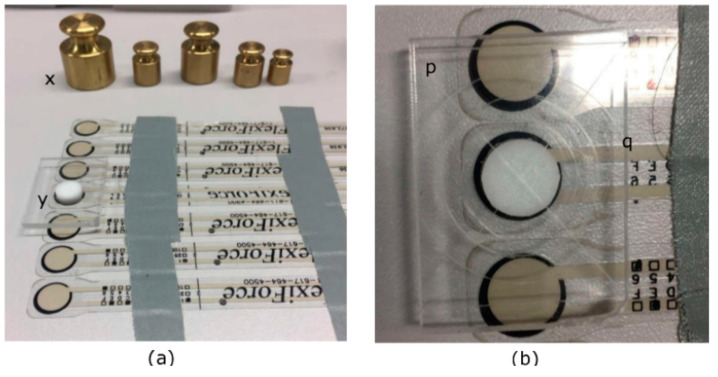
Calibration procedure for FlexiForce^®^ sensors (**a**) placement of the Teflon peg (white) and the Perspex sheet (x: the set of standard weights, y: Teflon peg). (**b**) The demarcations of different weights on the sheet (p: Perspex sheet, q: demarcations).

All sensors used in this study were calibrated as per manufacturer’s recommendations. Standard weights were used for calibrating the FlexiForce^®^ sensors; a range of weights was positioned on the circular sensing area. The cross-sectional area of the standard weights increases with the weight, therefore, to concentrate the load completely on to the sensing area and to prevent the weights touching the black surrounding ring of the sensor, a circular Teflon peg (9.53 mm diameter, 3 mm height, 0.442 g weight) was utilized as shown in Figure 2a. To avoid the misalignment of the weights, a Perspex sheet (25 mm × 35 mm × 3 mm) with demarcations of the base of standard weights 20 g, 50 g, 100 g (Figure 2b) was used. These demarcations would ensure that the weights are concentrically aligned with the sensor providing better repeatability to every sensor calibration. Table 1 shows the calibration points used by placing weights on the Perspex sheet. Although the manufacturer recommended only three calibration data points, six data points were used in this study for better accuracy (Table 1). Table 1 includes the weights which were used in the calibration process, along with the constant weight (Perspex sheet and Teflon peg) and this total weight was used to calculate the normal force applied on the sensor due to gravity (N). Then this perpendicular force was divided by the sensing area to calculate the pressure applied on to the sensor in N/mm^2^, which was then converted to mmHg. According to the manufacturer, once the sensors are calibrated, it does not require repeated calibrations unless the sensors have been damaged or bent.

AMI air-pack sensors were calibrated using a water column according to the manufacturer guidelines to 1 mmHg variation between displayed and calculated pressure value which is within the range of acceptable accuracy for the sensors.

PicoPress^®^ sensors were not calibrated, as the manufacturer has stated that it is not required to be calibrated before use [26]. However, before the use of the unit for measurement, a plunger which is connected to the micropump inside the main unit, is used to set the “zero pressure” measurement of the device. The plunger should be fully pulled out until “zero pressure” appears, and then has to be pushed back to adjust for the zero calibration. This calibration step ensures that the sensor measurements are recorded from zero pressure.

### 2.3. Validation against the Manometer

All the sensors used in this study were validated against a vertical liquid column manometer (VLCM), as the manometer is considered as the gold standard of pressure measurement by clinicians. This would enable the sensor behaviour to be studied and provide an extra validation step for the sensor calibration.

**Figure 3:**
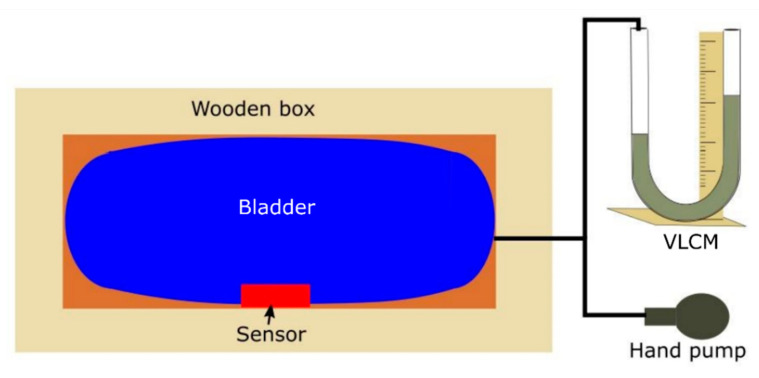
Schematic diagram for the experimental setup for validation of sensor readings against the vertical liquid column manometer (VLCM).

The validation of the sensors was carried out using a VLCM TJ600, measuring range 0−130 mbar, filled with Volt 1S manometer liquid of 1.86 g cm^−3^ density (KIMO Instruments, Kent, UK). A schematic of the test apparatus used in the study is given in Figure 3. The sensors were individually positioned inside the wooden box between the bladder and a side of the box. The bladder was then inflated by a hand-operated air-pump and the pressure inside the bladder was measured with the VLCM connected in series with the air-pump. The principle behind this set up was to apply pressure onto the sensor surface due to the expansion of the bladder [27]. While the pressure inside the bladder is equivalent to the external pressure applied on to the sensor, to avoid any error which could be caused due to the deformation of the bladder at its edges or any irregularity triggered by the inflation of the bladder, it was decided to position the sensor in the middle of the bladder.

Hence, a uniform pressure was applied onto the sensor surface due to the inflation of the bladder. The bladder was inflated in steps of 10 mbar (7.5 mmHg) and 2 mbar (1.5 mmHg), starting from 0 mbar (0 mmHg) up to 115 mbar (86.26 mmHg) and 80 mbar (60 mmHg) respectively while the corresponding pressure readings of the sensor were recorded for the sensors. For FlexiForce^®^ sensors, pressure readings of the sensor were recorded using the CEBO MSA DAQ lite 1.0 software (data acquisition mode of the CEBO software), and for both AMI air-pack and PicoPress^®^, the sensor pressure readings were directly recorded from the main unit display, the readings were taken after allowing the pressure to stabilize. The above procedure was repeated three times for each sensor, and the average pressure value was calculated for the subsequent calculations of the repeatability error and the non-linearity error. However, to calculate the hysteresis error, a separate test was conducted where the sensors were consecutively loaded and unloaded for each input pressure value and repeated for three cycles for each sensor. During the repeatability test, the four sensors of each batch were randomly subjected to each inflation cycle.

### 2.4. Evaluation of Sensor Performance

It was important to quantify the repeatability error for each sensor, as the use in compression therapy requires frequent, periodic pressure measurements using the same sensor. Generally, the repeatability error is used to quantify the ability of a measurement system to provide the same result for repeated measurements [25]. In general, sensor performances are quantified by nonlinearity error, repeatability error, and hysteresis error. Normally these error values for a sensor are quantified by the maximum error expressed as a percentage of the full span [28]. Hence, during the experiment, sensor readings were taken at least three (three repeated cycles) times for each input pressure value over a measurement range of 0–90 mmHg. In general, repeatability error for a sensor is quantified in terms of the maximum difference between two calibration cycles and expressed as a percentage of the full scale [29], or using 95% of the deviation range as expressed in Equation (1) [25,30,31]. However, in this research, repeatability error calculations were slightly altered to account for the biased evaluations that may occur due to outlier data points, as the maximum error might be caused by an outlier in the data set [25]. Thus, in this study, Equation (1) was adopted with a slight modification, where instead of quantifying it as 95% of the deviation range of maximum error, the average repeatability error over the sensor full span (using all the measurement points) plus the 95% confidence interval (CI) of the mean of the repeatability error was used [25]. The 95% CI was calculated using the t-value, assuming repeatability error over the full span of the sensor fit into a normal distribution. The t-value for larger data sets will be 1.96, however, it is larger than 1.96 for smaller data sets. The current study for the sensor evaluation includes smaller data sets (12 and 39 data points), hence the t-distribution was used for the CI calculation. Thus, the repeatability error for the sensors could be calculated as expressed in Equation (2); the repeatability error for all the sensors was calculated accordingly. The non-linearity error is defined as the error which occurs as a result of assuming a linear relationship between the input and the output over the working range [32]. The non-linearity error is often defined as a maximum non-linearity which could lead to incorrect information due to an outlier of data [30]. Hence, in this study, the non-linearity error was calculated for the full span as defined in Equation (3), by using the average of the non-linearity error in each data point over the full span plus the 95% CI which was calculated using the t distribution for the smaller data sets. In calculating hysteresis error, separate tests were carried out having consecutive loading and unloading cycles repeated three times for each sensor and the average readings for each input pressure were recorded to avoid any error caused by an outlier data set. Hence, in the same way, the hysteresis error for the sensors was calculated using the Equation (4). Accuracy of a measurement system can be defined as the extent to which the readings provided by the system might be wrong and can be quantified by summing up all the possible errors that are likely to occur [25,32]. The total error can be summed up using the general equation given as Equation (5).

(1) $$Repeatability\;error=\left(\frac{1.96\times SD\left(output\right)}{FS\;\left(output\right)}\times 100\right)$$

(2) $$Repeatability\;error=average\;\left(\frac{1.96\times SD\left(output\right)}{FS\;\left(output\right)}\times 100\right)+\left[t\times SE\;\left(\frac{1.96\times SD\left(output\right)}{FS\;\left(output\right)}\times 100\right)\right]$$

(3) $$non\;linearity\;error=average\;\left(\frac{\left|actual\;output-ideal\;output\right|}{FS\;\left(output\right)}\times 100\right)+\left[t\times SE\;\left(\frac{\left|actual\;output-ideal\;output\right|}{FS\;\left(output\right)}\times 100\right)\right]$$

(4) $$Hysteresis\;error=average\;\left(\frac{\left|loading\;output-unloading\;output\right|}{FS\;\left(output\right)}\times 100\right)+\left[t\times SE\;\left(\frac{\left|loading\;output-unloading\;output\right|}{FS\;\left(output\right)}\times 100\right)\right]$$

where; *SD*—standard deviation, *FS*—full span (maximum output value recorded during the evaluation), *SE*—standard error ($SE=\frac{SD}{\surd n}$), where n is equal to the number of readings, *t*—t value from the ‘t-distribution’, for a 95% confidence interval (with n-1 degree of freedom).

(5) $$\Delta Z=\surd Z\Delta A_{i}$$

where; ΔZ = total error, $\Delta A_{i}$ = error.

## 3. Results and Discussion

### 3.1. FlexiForce^®^ Sensor Evaluation

**Figure 4:**
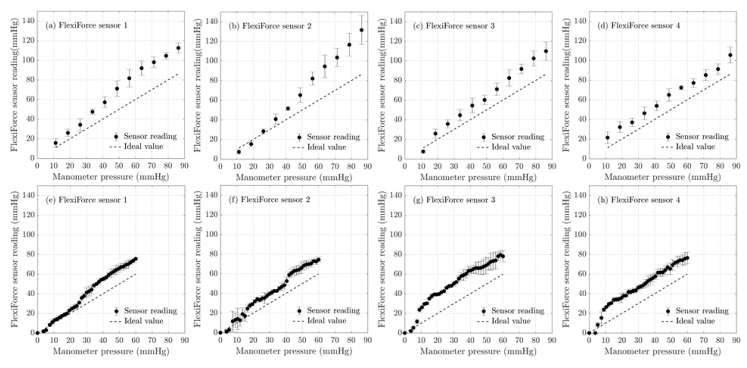
The results obtained using the bladder test: validation of FlexiForce^®^ sensors readings against the manometer reading (standard weight calibrated). The error bars represent the 95% confidence interval. (**a**–**d**) represent the FlexiForce^®^ sensors of batch one and the (**e**–**h**) represents the FlexiForce^®^ sensors of batch two.

Figure 4 shows the experimental results obtained for the eight FlexiForce^®^ sensors from two batches, and Figure 4a–d shows the sensor readings of the corresponding sensors of the first batch and e–h corresponds to the sensors of the second batch. Since the sensors were calibrated in mmHg, the sensor readings were recorded in mmHg in the data acquisition mode of the CEBO MSA software. As it is evident from Figure 4a–d, the pressure sensor readings have deviated greatly from the ideal line, making it clear that the sensors display a huge error in their reading. It was also evident that there is a large variation of the measured pressure by sensors from the ideal line in the high-pressure regions and the measured sensor values have overestimated the pressure readings on average by more than 15 mmHg. This margin of overestimation of pressure values could draw wrong conclusions in the research in measuring active and passive pressure.

The bladder was inflated in steps of 10 mbars (7.5 mmHg), starting from a pressure of 0 mbar (0 mmHg) up to a pressure of 115 mbar (86.26 mmHg) for the first set of sensors and later it was inflated in steps of 2 mbars (1.5 mmHg), starting from a pressure of 0 mbar (0 mmHg) up to a pressure of 80 mbar (60 mmHg) to obtain higher resolution by using more data points in the validation step. Then, the repeatability error was calculated for these sensors as per Equation (2) and the results were tabulated in Table 2.

The manufacturer specified repeatability error and the nonlinearity error for FlexiForce^®^ sensors are less than +/−2.5% FS and less than +/−3% FS [33] respectively. The measured repeatability error for the first batch of sensors was 7.6% FS (on average), while for the second batch it was 4.3% FS (on average). Both measured repeatability error values are higher than the manufacturer’s specified values. However, the pressures recorded with the second batch of sensors were significantly higher compared to the manometer readings as can be seen from Figure 4e–h. The dashed line in Figure 4e–h. shows the correct reading that the sensor should record to match with the manometer reading, which clearly indicates that the sensors are over-estimating the pressure values (on average 1.5 times the manometer reading). It was also evident from Figure 4e–h that the relationship between the manometer reading and the sensor reading could hardly be deduced to a linear relationship. The calibration curve for the manometer and the sensors has shown a quadratic relationship, which would make it difficult to have a detailed analysis. Hence, calculating a non-linearity error for this batch of sensors would be invalid in this context.

The evaluation of the sensors also demonstrated that the measured nonlinearity error for the first batch of FlexiForce^®^ sensors was 17.7 % FS (on average), which is significantly higher (more than 5 times) than the manufacturer’s specified value. It was also evident that individual FlexiForce^®^ sensors exhibited significant variations (maximum standard deviation of 10 mmHg), which could lead to errors when measuring pressure at different points simultaneously. It could be also noted that a significant variation from batch to batch among these FlexiForce^®^ sensors was detected in terms of nonlinearity and repeatability errors. This could lead to errors in measuring pressures simultaneously, using multiple sensors for compression therapy applications. The total error for the first batch of sensors was as high as 19% FS on average (excluding the hysteresis error), hence the accuracy is less than ±22 mmHg. Since both the repeatability and non-linearity error percentages obtained were significantly higher than the manufacturer’s specified values and the batch to batch variation of the sensors was also high, it was concluded that these sensors would be difficult for the clinicians to use in the treatment of venous insufficiency; therefore, the hysteresis error was not calculated for these sensors.

### 3.2. AMI Air-Pack Sensor Evaluation

**Figure 5:**
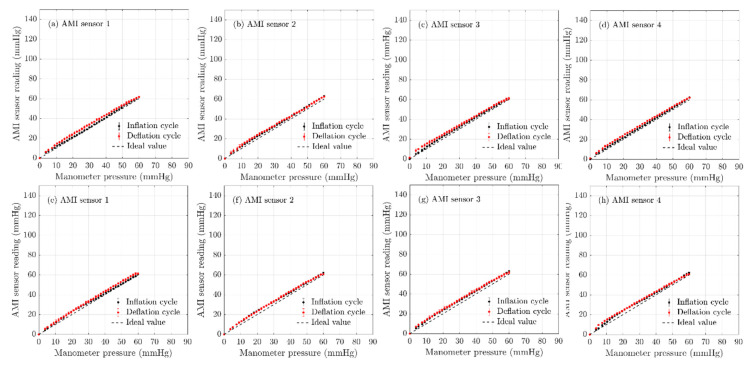
The results obtained using the bladder test: the validation of AMI air-pack sensor readings against the manometer reading. The error bars represent the 95% confidence interval. (**a**–**d**) represent the AMI air-pack sensors of batch one and the (**e**–**h**) represents the AMI air-pack sensors of batch two.

Figure 5 shows the experimental results obtained for the eight AMI air-pack sensors from two batches. Figure 5a–d shows the sensor readings of the sensors of the first batch and Figure 5e–h corresponds to the sensor readings of the second batch.

The bladder was inflated in steps of 2.0 mbars (1.5 mmHg), starting from a pressure of 0mbar (0.0 mmHg) up to a pressure of 80.0 mbar (60.0 mmHg). After the bladder was inflated up to a pressure of 80.0 mbar (60.0 mmHg), it was deflated in steps of 2.0 mbars (1.5 mmHg) to determine the hysteresis of the sensors. This enabled a relationship between the air-pack sensor (display value of the control unit) and the manometer reading to be determined. As shown in Figure 5, the linear relationship between the AMI air-pack sensor reading and the manometer reading was evident. In comparison with the FlexiForce^®^ sensors, the AMI air-pack sensors demonstrated a higher accuracy, i.e., the error between the sensor and manometer readings were less than 2.5 mmHg, with the AMI pressure measurement system.

The repeatability errors for the AMI air-pack sensors were also calculated using Equation (2), and the results are tabulated in Table 3. The repeatability error for AMI air-pack sensors was 2.5% FS on average compared to the FlexiForce^®^ sensors 7.6% FS and 4.3% FS. Also, the air-pack sensors showed a low repeatability error between sensors (Table 3) (maximum standard deviation of 2% FS, which is less than 1.5 mmHg). The data in Table 3 demonstrates that the hysteresis error for these sensors was also low (less than 3% FS). As the VLCM is considered to be the gold standard for the pressure measurement by the medical community and medical device manufacturers, the linear relationship obtained can be used for interface pressure measurement in compression therapy.

It could be noted from the results listed in Table 3, the AMI air-pack sensors have shown a very low non-linearity error (%) of less than 1.5% FS (less than 1.5 mmHg). This shows that the AMI air-pack sensors are suitable for accurate interface pressure measurement in compression therapy. The results given in Table 3 show an average accuracy of ±2.2 mmHg for the AMI air-pack sensors. Although the manufacturer’s specified accuracy for the sensor is ±1.5 mmHg at 23 °C, the test data showed that actual accuracy is slightly lower than the manufacturer’s specified value. This might be due to the fact that the sensors were not tested in a conditioned atmosphere.

However, comparatively, all the tested AMI air-pack sensors showed a close approximation to the manufacturer’s specified accuracy. When measuring skin interface pressure, the sensors would be in contact with the human body, where the temperature is around 37 °C. Since the AMI air-pack sensors are calibrated using 20–30 °C warm water, which is less than the human body temperature, one could argue that a measurement error is evident, which can be explained with “Combined Gas Law”. However, according to the literature, the AMI air-pack sensors have reportedly shown a very low sensitivity to a larger temperature change, for example, 0.1 kPa (i.e., 0.75 mmHg) change of pressure reading to 40 °C change of temperature [34].

### 3.3. PicoPress^®^ Sensor Evaluation

**Figure 6:**
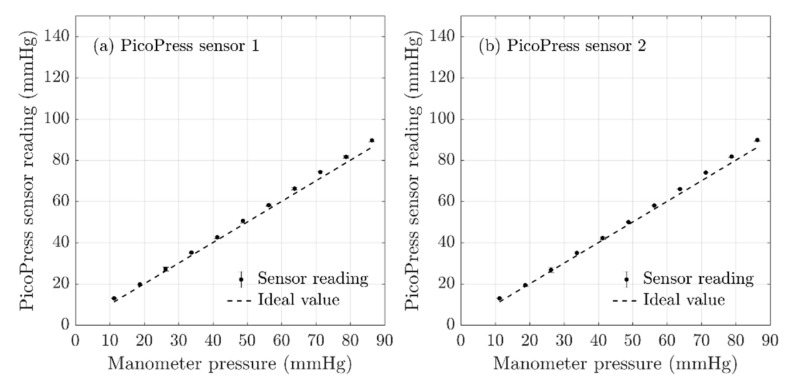
The results obtained using the bladder test, validation of PicoPress^®^ sensors readings against the VLCM reading. The error bars represent the 95% confidence interval (**a**), (**b**) represents the two PicoPress^®^ sensors.

The same procedure described in Section 2.3 was carried out with two PicoPress^®^ sensors and the obtained results are shown in Figure 6. The figure shows that the PicoPress^®^ sensor readings demonstrated a very good agreement with the VLCM data, with a linear relationship having an average non-linearity error of less than 0.7% FS. The calculated non-linearity errors and the repeatability errors are tabulated in Table 4. The PicoPress^®^ sensors showed the best linear relationship out of all three pressure measurement systems evaluated, having the error between sensor reading and manometer reading less than 2 mmHg on average. According to the repeatability errors tabulated in Table 4, the error values had the lowest when compared to the other two pressure measuring systems studied. The variance of the readings between the sensors was also minimum in this pressure measurement system (the standard deviation was less than 0.07 mmHg), which makes it most suitable in measuring pressures at different points simultaneously. Hence, the PicoPress^®^ pressure measuring system had demonstrated that it was the most reliable pressure measurement system for compression therapy.

The sensor dimensions play an important role in the selection of the interface pressure measuring system for compression therapy. Although PicoPress^®^ sensors provided the most accurate results in comparison to the other available interface measurement systems, its applications in measuring interface pressure of small sensing areas (such as pressure exerted in the thumb area) are limited due to its large probe size (50 mm in diameter).

Apart from the above analysis, it should also be noted that the sensor choice would be affected by the clinical practice of measuring the compression pressure. The FlexiForce^®^ sensors tend to provide faulty readings in measuring curvatures less than 32 mm [10] due to the bending of the sensor. Hence, there is a tendency that these could provide faulty pressures in bony prominence areas of the leg. In clinical practice, it would require the measurement unit to be portable and easy to carry, which will be convenient for the clinician. In this context, the PicoPress^®^ and FlexiForce^®^ inherit a smaller measurement unit whereas the AMI has a considerably larger measurement unit. However, all three sensors have thin and flexible sensing probes which makes them more suitable in the measurement of compression pressure under clinical conditions.

## 4. Conclusions

This research was focused on evaluating three commercially available interface pressure measurement systems on their repeatability, accuracy, and linearity for compression therapy applications. The selection of the interface pressure measuring system for compression therapy should have the following characteristics which are the initial requirements to reduce the chance of error occurrence. The sensor dimensions to be compatible with the interface surface, higher repeatability of the sensor data, good linearity of the sensor data, and low hysteresis. As described above, among the available pressure measurement systems it was evident that even though the FlexiForce^®^ sensors were the most compatible sensor with respect to the dimensions and the thinner nature of the sensor, for this application its performance was not accurate enough compared to the other two tested interface pressure measurement systems. The repeatability errors as high as 10% FS were observed during the evaluation of FlexiForce^®^ sensors against the manometer. It was also observed that obtaining a linear relationship between the manometer reading and the sensor reading led to higher non-linearity errors, and the data had to be fitted on to a higher degree polynomial in order to obtain a good fit. This is a disadvantage as it would require a complex relationship that would make it challenging for the further procession of the data. Also, a significant variation between the FlexiForce^®^ sensors was observed which could result in errors when measuring interface pressure simultaneously at different positions as demanded by the research. The validation of the sensors against the manometer showed that FlexiForce^®^ sensors recorded overstated pressure values most of the time, as high as 15 mmHg.

In comparing the repeatability, the best results were noted for PicoPress^®^ sensors with the repeatability error as low as 0.73% FS. The study of the three pressure measurement systems showed that the most reliable results recorded during the validation process against the manometer were obtained with PicoPress^®^ sensors. However, PicoPress^®^ sensors have a relatively large sensing area of 1964 mm^2^ (circular geometry of a diameter of 50 mm), which makes it disadvantageous to be used in smaller contact area surfaces. AMI air-pack sensors with a sensing area of 20 mm diameter showed a maximum repeatability error of 3.6% FS, while on average it was 2.5% FS, which is low, and the measured data were reproducible. AMI air-pack sensors also showed very good linearity, with an average non-linearity error of 1.5% FS. The average hysteresis error recorded by the AMI air-pack sensors was 2.2% FS, which is also low, and it assured that the recorded pressure values are least affected by the sensor hysteresis.

As per the main findings of the research, pneumatic-based interface pressure measurement systems are accurate and generate reproducible pressure readings for the interface pressure measurement in the compression therapy compared to the piezoresistive force sensors. Thus, it is recommended to use pneumatic based pressure measurement systems for compression therapy applications.

## Figures and Tables

**Table 1:** The calibration points used with FlexiForce^®^ sensors using standard weight.

Constant (Peg and Perspex Sheet) Weight (g)	Standard Weight (g)	Total Weight (g)	Force (N)	Pressure (N/mm^2^)	Pressure (mmHg)
3.463	20	23.463	0.2301	0.00324	24.31
3.463	40	43.463	0.4363	0.006	45.03
3.463	50	53.463	0.5343	0.00739	55.39
3.463	70	73.463	0.7205	0.01015	76.11
3.463	80	83.463	0.8185	0.01153	86.48
3.463	100	103.463	1.0147	0.01429	107.2

**Table 2:** Repeatability and non-linearity error (%) for FlexiForce^®^ sensors of batch 1 and 2.

Sensor	Nonlinearity Error (%)	Repeatability Error (%)
Batch 1		
Sensor 1	15.82	7.49
Sensor 2	20.86	9.51
Sensor 3	20.19	6.87
Sensor 4	13.85	6.59
Batch 2		
Sensor 1		2.92
Sensor 2		4.97
Sensor 3		4.98
Sensor 4		4.16

**Table 3:** Repeatability and non-linearity error (%) for AMI air-pack sensors of batch 1 and 2.

Sensor	Nonlinearity Error (%)	Repeatability Error (%)	Hysteresis Error (%)	Accuracy (mmHg)
Batch 1				
Sensor 1	1.22	3.62	2.37	2.67
Sensor 2	0.52	1.48	1.60	1.41
Sensor 3	0.64	2.60	3.39	2.65
Sensor 4	0.31	2.00	3.18	2.35
Batch 2				
Sensor 1	1.17	2.08	2.69	2.18
Sensor 2	0.97	2.08	0.91	1.53
Sensor 3	0.88	3.07	1.39	2.19
Sensor 4	1.27	2.67	1.91	2.19

**Table 4:** Repeatability and non-linearity error (%) for PicoPress^®^ sensors.

Sensor	Nonlinearity Error (%)	Repeatability Error (%)
Sensor 1	0.57	0.84
Sensor 2	0.81	0.73

## References

[1] Nandasiri G.K., Ianakiev A., Dias T. (2020). Hyperelastic Properties of Platinum Cured Silicones and its Applications in Active Compression. Polymers.

[2] Kakkos S.K., Timpilis M., Patrinos P., Nikolakopoulos K.M., Papageorgopoulou C.P., Kouri A.K., Ntouvas I., Papadoulas S.I., Lampropoulos G.C., Tsolakis I.A. (2018). Acute Effects of Graduated Elastic Compression Stockings in Patients with Symptomatic Varicose Veins: A Randomised Double Blind Placebo Controlled Trial. J. Vasc. Surg..

[3] Attaran R.R., Chaar C.I.O. (2017). Compression Therapy in Venous Disease. Curr. Manag. Venous Dis..

[4] DePopas E., Brown M. (2018). Varicose Veins and Lower Extremity Venous Insufficiency. Semin. Interv. Radiol..

[5] Yamany A., Hamdy B. (2016). Effect of sequential pneumatic compression therapy on venous blood velocity, refilling time, pain and quality of life in women with varicose veins: A randomized control study. J. Phys. Ther. Sci..

[6] Zhao S., Liu R., Fei C., Guan D. (2019). Dynamic Interface Pressure Monitoring System for the Morphological Pressure Mapping of Intermittent Pneumatic Compression Therapy. Sensors.

[7] Flaud P., Bassez S., Counord J.-L. (2010). Comparative In Vitro Study of Three Interface Pressure Sensors Used to Evaluate Medical Compression Hosiery. Dermatol. Surg..

[8] Partsch B., Partsch H. (2005). Calf compression pressure required to achieve venous closure from supine to standing positions. J. Vasc. Surg..

[9] Milic D.J., Zivic S.S., Bogdanovic D.C., Jovanovic M.M., Jankovic R.J., Milosevic Z.D., Stamenkovic D.M., Trenkic M.S. (2010). The influence of different sub-bandage pressure values on venous leg ulcers healing when treated with compression therapy. J. Vasc. Surg..

[10] Ferguson-Pell M., Hagisawa S., Bain D. (2000). Evaluation of a sensor for low interface pressure applications. Med. Eng. Phys..

[11] Brophy-Williams N., Driller M., Halson S.L., Fell J., Shing C.M. (2013). Evaluating the Kikuhime pressure monitor for use with sports compression clothing. Sports Eng..

[12] Mosti G., Rossari S. (2008). The importance of measuring sub bandage pressure and presentation of new measuring device. Acta Vulnol.

[13] Damstra R.J., Partsch H. (2013). Prospective, randomized, controlled trial comparing the effectiveness of adjustable compression Velcro wraps versus inelastic multicomponent compression bandages in the initial treatment of leg lymphedema. J. Vasc. Surg. Venous Lymphat. Disord..

[14] Luo S., Wang J., Shi H., Yao X. (2015). A novel approach to characterize dynamic pressure on lower limb wearing compression cycling shorts. J. Text. Inst..

[15] Liu R., Kwok Y.L., Li Y., Lao T.T.H., Zhang X., Dai X.Q. (2005). Objective Evaluation of Skin Pressure Distribution of Graduated Elastic Compression Stockings. Dermatol. Surg..

[16] Liu R., Kwok Y.L., Li J.-S., Lao T.T., Zhang X. (2007). Skin pressure profiles and variations with body postural changes beneath medical elastic compression stockings. Int. J. Dermatol..

[17] Li Y., Dai D.X. (2006). Biomechanical Engineering of Textiles and Clothing.

[18] Lai C.H., Li-Tsang C.W. (2009). Validation of the Pliance X System in measuring interface pressure generated by pressure garment. Burns.

[19] Partsch H., Mosti G. (2010). Comparison of three portable instruments to measure compression pressure. Int. Angiol..

[20] Al Khaburi J., Dehghani-Sanij A.A., Nelson E.A., Hutchinson J. Pressure mapping bandage prototype: Development and testing. Proceedings of the 2012 International Conference on Biomedical Engineering (ICoBE).

[21] Al Khaburi J., Nelson E.A., Hutchinson J., Dehghani-Sanij A.A. (2011). Impact of multilayered compression bandages on sub-bandage interface pressure: A model. Phlebol. J. Venous Dis..

[22] Parmar S., Khodasevych I.E., Troynikov O. (2017). Evaluation of Flexible Force Sensors for Pressure Monitoring in Treatment of Chronic Venous Disorders. Sensors.

[23] Al Khaburi J., Dehghani-Sanij A.A., Nelson E.A., Hutchinson J. Measurement of interface pressure applied by medical compression bandages. Proceedings of the 2011 IEEE International Conference on Mechatronics and Automation.

[24] Chi Y.-W., Tseng K.-H., Li R., Pan T. (2017). Comparison of piezoresistive sensor to PicoPress^®^ in in-vitro interface pressure measurement. Phlebol. J. Venous Dis..

[25] Al Khaburi J.A.J. (2010). Pressure Mapping of Medical Compression Bandages Used for Venous Leg Ulcer Treatment.

[26] Microlab Electronica PicoPress^®^ COMPRESSION Measurement System. http://www.microlabitalia.it/wfolder/filescasehistory/file/11.pdf.

[27] Hakala T.T.M., Puolakka A., Nousiainen P., Vuorela T., Vanhala J. (2017). Application of air bladders for medical compression hosieries. Text. Res. J..

[28] Bentley J.P. (2005). Principles of Measurement Systems.

[29] Fraden J. (2004). Handbook of Modern Sensors: Physics, Designs, and Applications.

[30] Dunn P.F., Davis M.P. (2017). Measurement and Data Analysis for Engineering and Science.

[31] Niu Z., Zhao T., Zhao Y., Hu Q., Ding S. (2017). Design and Analysis of the Measurement Characteristics of a Bidirectional-Decoupling Over-Constrained Six-Dimensional Parallel-Mechanism Force Sensor. Sensors.

[32] Bolton W. (1996). Measurement and Instrumentation Systems.

[33] Tekscan^®^ (2018). FlexiForce^TM^ Standard Model A201. https://www.tekscan.com/products-solutions/force-sensors/a201.

[34] AMI Techno (2009). Air-Pack Type Contact Surface Pressure Measurement System. https://www.ami-tec.co.jp/pdf/e_20sessyoku_20siyou.pdf.

